# Deeping in the Role of the MAP-Kinases Interacting Kinases (MNKs) in Cancer

**DOI:** 10.3390/ijms21082967

**Published:** 2020-04-23

**Authors:** Celia Pinto-Díez, Raquel Ferreras-Martín, Rebeca Carrión-Marchante, Víctor M. González, María Elena Martín

**Affiliations:** Grupo de Aptámeros, Servicio de Bioquímica-Investigación, IRYCIS-Hospital Ramón y Cajal, Madrid, Ctra. Colmenar Km. 9100, 28034 Madrid, Spain; celia.pinto@aptusbiotech.com (C.P.-D.); raquel.ferreras@hrc.es (R.F.-M.); rebeca.carrion@hrc.es (R.C.-M.); victor.m.gonzalez@hrc.es (V.M.G.)

**Keywords:** antitumor drug, cancer, eIF4E, metastasis, MNK, therapy

## Abstract

The mitogen-activated protein kinase (MAPK)-interacting kinases (MNKs) are involved in oncogenic transformation and can promote metastasis and tumor progression. In human cells, there are four MNKs isoforms (MNK1a/b and MNK2a/b), derived from two genes by alternative splicing. These kinases play an important role controlling the expression of specific proteins involved in cell cycle, cell survival and cell motility via eukaryotic initiation factor 4E (eIF4E) regulation, but also through other substrates such as heterogeneous nuclear ribonucleoprotein A1, polypyrimidine tract-binding protein-associated splicing factor and Sprouty 2. In this review, we provide an overview of the role of MNK in human cancers, describing the studies conducted to date to elucidate the mechanism involved in the action of MNKs, as well as the development of MNK inhibitors in different hematological cancers and solid tumors.

## 1. Introduction

**Figure 1:**
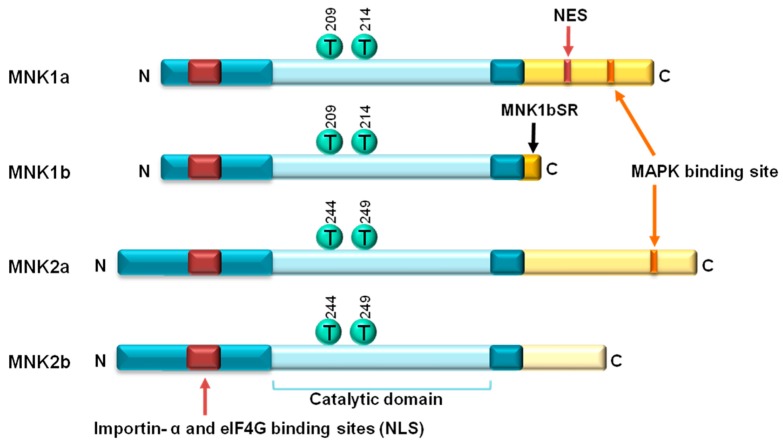
General scheme of human MNKs. All MNKs have a nuclear localization sequence (NLS) and threonines of the catalytic domain, while only MN1a has the nuclear export sequence (NES) and only MN1a and MN2a possess the MAPK binding domain.

The Mitogen-activated protein kinase (MAPK)-interacting kinases (MNKs), serine/threonine kinases encoded on two genes (*MKNK1 and MKNK2*), were identified simultaneously in mice [1] and in humans [2] in 1997. In humans, each gene gives rise to two isoforms from an alternatively spliced transcript, named MNK1a/MNK1b and MNK2a/MNK2b [3,4] (Figure 1). All the MNK isoforms have a nuclear localization signal (NLS) in the N-terminal region that mediates the binding with α-importin protein, which allows the kinase to enter into the nucleus. MNK1a and MNK2a are both primarily cytoplasmic, whereas MNK1b and MNK2b localize partially to the nucleus. MNK1a contains a nuclear export signal (NES) that ensures its cytoplasmic localization [5], whereas MNK1b lacks this NES, being cytoplasmic and nuclear [4]. MNK2a and MNK2b lack this NES but other mechanisms ensure the cytoplasmic localization of MNK2a, whereas MNK2b is largely nuclear [5,6]. The N-terminal region also contains the binding domain to the eukaryotic initiation factor 4G (eIF4G), protein that is part of the eukaryotic initiation factor 4F (eIF4F). The eIF4F complex consists of three factors: eIF4E, eIF4A, an ATP-dependent RNA helicase, and eIF4G, a scaffold protein for the assembly of eIF4E and eIF4A. MNKs efficiently phosphorylate their substrate eIF4E when both proteins are bound to the eIF4G scaffold protein, which facilitates their nearness [7]. 

MNK1a and MNK2a present a canonical MAP kinase binding motif in the C-terminal region, although their sequences differ slightly (LARKR and LAQRR sequence, respectively) such that MNK1a binds both extracellular signal-regulated kinases (ERK) 1/2 and p38 kinases, while MNK2a associates only with ERK1/2 [1]. However, MNK1b and MNK2b, truncated isoforms with distinct C-terminal regions to the longer isoforms, lack the MAPK binding domain and seem to be independent of the upstream kinases (ERK1/2 and p38 MAP kinases) [4]. In spite of the presence or not of the MAPK kinase binding motif, the four isoforms present different basal activities. The basal activity of MNK2a is higher than MNK1a due to the sustained association of MNK2a with ERK1/2 whereas MNK1a has low basal activity but is responsive to the ERK1/2 and p38 activation [8]. MNK1b and MNK2b show to have high and low basal activity, respectively [4,6,9]. 

The central region of MNK1 and MNK2 corresponds to the catalytic domain of the protein with a similarity in the amino acid sequence of 78% between them. The active sites are highly conserved, with two threonine residues (209 and 214 in MNK1, and 244 and 249 in MNK2) that make up the activation loop of the kinase activity. These threonines of the activation loop are followed by prolines that function as phosphorylable residues, so that they are susceptible to being phosphorylated by MAPKs, characteristic that MNKs have in common with MAPK-activated protein kinases (MK2, MK3 (or pK3) and MK5), with the p90S6 protein kinase (RSK) and with the mitogen-activated and stress-activated protein kinase (MSK). However, they also have particularities in the catalytic domains. MNKs have a DFD (Asp-Phe-Asp) motif that marks the beginning of the activation loop, instead of DFG (Asp-Phe-Gly) that other kinases have. In addition, the domains of MNK1 and MNK2 contain two insertions, one located between the DFD motif and the activation loop and another next to the APE motif (Ala-Pro-Glu) [10]. Studies of the three-dimensional structures of MNK1 and MNK2 indicate that the activation loop of MNK2 acquires an unusual open conformation and the DFD motif interferes with ATP binding [10]. On the other hand, the activation loop of MNK1 is self-inhibiting, since it contains a Phe230 that moves the Phe192 from the DFD to the ATP binding site, preventing its binding [11]. 

In mice, only MNK1a and MNK2a isoforms have been identified and both proteins are expressed in all adult tissues, except in the brain where MNK2 levels are very low. In comparison with the rest of tissues, the expression of both proteins is rather abundant in skeletal muscle [1]. In humans, the expression of MNK1a is higher in the liver, pancreas, heart and placenta. MNK1b is expressed in all tissues studied at similar extent except skeletal muscle, where its levels are low [4]. MNK2 isoforms have a wide distribution in all the tissues studied, except in brain and heart where their levels are low. However, like MNK1a, the levels of both isoforms, MNK2a and MNK2b, are higher in the pancreas [3].

## 2. MNKs Substrates

The only well-characterized substrate for MNK1 is the eukaryotic initiation factor 4E (eIF4E). eIF4E was first identified as substrate of the MNKs both in vitro and in vivo [1,12]. Later, studies in vivo with knock-out mice for *Mnk1 and Mnk2*, in which phosphorylation of Ser209 was not detected under any type of activation, corroborate that MNK1/2 are the only kinases for eIF4E [13]. The eIF4E is part of the eIF4F complex and specifically binds to the 5′ cap structure of the eukaryotic cytoplasmic mRNAs and subsequently recruits the mRNA to the ribosome [reviewed in [14,15]]. 

The effect of eIF4E phosphorylation on eIF4F activity is controversial. Initially, it was thought that phosphorylation of Ser209 of eIF4E by MNKs, which normally occurs in response to agents that activate protein synthesis, increased its affinity for the cap of mRNAs to form a more stable eIF4F complex. However, several reports support that eIF4E phosphorylation markedly reduces its affinity for capped mRNA [16,17,18]. In the nucleus, eIF4E promotes nuclear export of a subset of specific mRNAs [19]. Borden’s laboratory has demonstrated that the phosphorylation of nuclear eIF4E seems to be an important step in the control of the mRNA transport [20]. Consistently, several findings support that eIF4E phosphorylation can play a role in the transport of cyclin D1 from the nucleus to the cytoplasm which drives to cell transformation.

**Figure 2:**
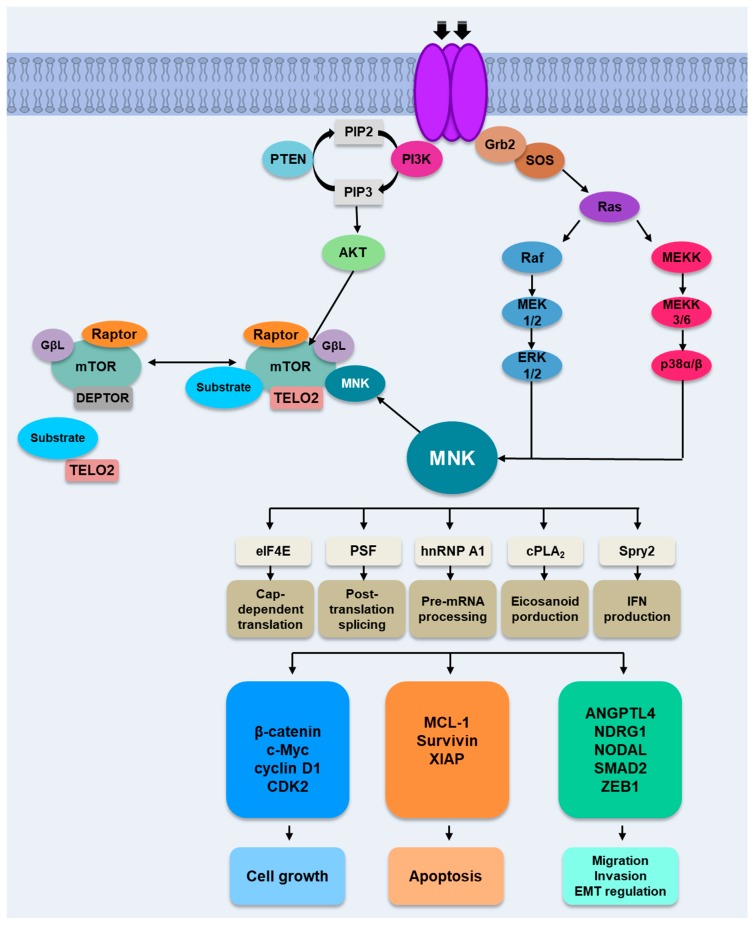
Mechanism of Action of MNKs. Activation of MNKs occurs through the activation of the Ras/Raf/ERK cell signaling pathway and p38 MAPK pathway. Likewise, the activation of the PI3K/AKT/mTOR pathway in response to growth factors, among others, stimulates the binding of MNK to mTORC1, regulating the formation of the mTORC1/TELO2/DDB1 complex. MNKs phosphorylate eIF4E and other substrates controlling the expression of specific proteins involved in cell growth, apoptosis and metastasis.

To date, other MNKs substrates, in addition to eIF4E, have been identified—hnRNP A1, PSF, cPLA_2_ and Spry2—but their relevance in vivo is not yet established (Figure 2). The Heterogeneous Nuclear Ribonucleoprotein A1 (hnRNP A1) is a very abundant nuclear protein that plays an important role in mRNA metabolism. Although it is a nuclear protein, it shuttles between the nucleus and the cytoplasm through the non-classic nuclear localization domain, called M9 motif. MNKs phosphorylate hnRNP A1 in Ser192 and Ser310 in response to T-cell activation. The inhibition of MNK activity with CGP57380 decreases the release of the tumor necrosis factor (TNF) α and the interleukins IL-6 and IL-1β in response to anisomycin (a p38 MAPK agonist) in human keratinocyte cultures [21] and causes a decrease in TNFα production by macrophages after treatment with multiple agonists of the Toll-like receptor (TLR) family [22]. MNK1 participates in the regulation of TNFα synthesis through phosphorylation of the hnRNP A1 protein, which decreases its ability to bind to AREs regions (3’UTR regions of mRNA rich in residues A and U) in the TNFα mRNA that causes derepression of its translation [23]. In addition, hnRNP A1 is phosphorylated in response to osmotic stress through p38 MAPK, so MNKs would also regulate the translation of specific mRNAs in cell stress response [23,24]. The Polypyrimidine tract-binding protein-associated Splicing Factor (PSF) is a nuclear protein involved in RNA transcription and processing. Together with p54nrb, another DNA/RNA binding protein, it forms a transcription/processing factor involved in multiple nuclear processes and in tumorigenesis [25]. It also regulates positively the translation of the *Myc* family of oncogenes, among other processes [26]. Buxade et al. identified PSF as a new intracellular substrate of MNK in vitro [27]. They identified two phosphorylation sites in PSF, Ser8 (preferably phosphorylated by MNK2) and Ser283. PSF interacts with mRNAs containing AREs and phosphorylation by MNK increases its binding to TNFα mRNA in vivo, although it does not affect the stability or nuclear/cytoplasmic localization of PSF or TNFα mRNA [27]. A more recent study has revealed the role of MNK in TNFα synthesis by controlling the abundance of its mRNA [28], although the involvement of PSF and/or hnRNP A1 has not been determined. The cytoplasmic phospholipase A2 (cPLA_2_) plays a key role in the production of eicosanoids that participate in immunity and inflammation processes. MNK1 phosphorylates cPLA_2_ in Ser727 in vitro [29], which is regulated by the p38 MAPK signaling pathway. This phosphorylation causes the activation of cPLA2, which releases arachidonic acid from glycerophospholipids for the production of eicosanoids. Sprouty (Spry) proteins are a group of membrane-associated proteins that suppress the activation and/or signaling of ERK. MNK1 phosphorylates Spry2 in Ser112 and Ser121 stabilizing Spry2 and lengthen its ability to inhibit ERK signaling [30].

## 3. MNK and Cancer

The relationship between eIF4E and cell growth control and neoplastic transformation was first published in 1990 [31]. These authors demonstrated that overexpression of eIF4E in the NIH3T3 cells inhibits the growth of agar colonies and produces tumors when inoculated into mice. In addition, inhibition of eIF4E reduces tumor growth and malignancy in experimental models [32]. The increased expression of eIF4E preferentially induces the translation of proteins involved in cancer such as vascular endothelial growth factor (VEGF) and fibroblast growth factor (FGF) that facilitate angiogenesis, Bcl-2 that participates in cell survival, metalloproteases (MMP) involved in invasion and c-Myc, cyclin D1, ornithine decarboxylase (ODC) and the human double minute 2 homolog (HDM2) that regulate cell growth [19,20,33,34,35,36].

It has been shown eIF4E overexpression in a variety of cancers including breast, bladder, colon, head and neck, kidney, lung, skin, ovarian and prostate compared to healthy tissues and its relationship with disease progression (reviewed in [14]). In addition, elevated levels of phosphorylated eIF4E have been found in human cancer tissues obtained from patients with lung, head, colorectal, and gastric cancers and primary pancreatic ductal adenocarcinoma [37,38]. Several studies established that the phosphorylation of eIF4E on Ser209 by MNK1/2 is an absolute requirement for the oncogenic action of eIF4E. The inhibition of MNK activity reduces colony formation in human breast cell lines [39]. On the other hand, overexpression of the oncogene *HMD2* in cancer cells is regulated by eIF4E, so that the overexpression of eIF4E promotes the export of the HDM2 mRNA in a MAP kinase- and MNK1-dependent manner [35]. In addition, Wendel et al. have shown that the overexpression of a constitutively active MNK1 diminishes the apoptosis and accelerates the development of tumors in an experimental model of mice while an inactive mutant reduces the development of these tumors [36]. Ueda et al. have demonstrated that the absence of MNK1/2 does not alter the normal development of mice, although it delays mouse tumor progress [40].

The activity of eIF4E is also regulated by its availability to participate in the initiation of translation through binding with 4E-BP proteins which form an inactive complex with eIF4E, inhibiting the binding thereof to eIF4G and thereby preventing the formation of the eIF4F complex required for initiating protein synthesis [41]. The complex 1 of the mammalian target kinase protein of rapamycin (mTORC1) regulates the assembly of the eIF4F complex through the phosphorylation of 4E-BPs, which involves the disassociation of eIF4E and the binding to eIF4G, where it becomes available for being phosphorylated by MNKs.

The PI3K/AKT/mTOR signaling cascade is among one of the most frequently deregulated mechanisms in cancer, often as a result of genetic alterations and/or mutations [42]. This pathway plays a key role in tumor cell proliferation, survival and development, and its deregulation is closely linked to tumorigenesis and to the sensitivity and resistance to cancer therapies. Growth factors, mitogens and cytokines activate the phosphatidylinositol-3 kinase (PI3K), which initiates a cascade of cellular events. The 3′ phosphoinositol-dependent kinase-1 (PDK1) activates the protein AKT (AKT enzyme, also called Protein Kinase B) which, by means of the inactivation of tumor suppressor complex 1 and 2 (TSC1/2), activates the mammalian target kinase protein of rapamycin (mTOR) complex 1, mTORC1. The activation of PDK1 and AKT by PI3K is negatively regulated by *PTEN*, a tumor suppressor gene which is usually mutated or silenced in human cancers [43,44]. The loss of the phosphatase and tensin homolog (PTEN) causes the activation of AKT and of mTORC1 signaling. mTORC1 phosphorylates the 4E-BPs and also promotes the activation of the kinase S6K which phosphorylates ribosomal protein S6 [45]. There are evidences suggesting a compensatory feedback mechanism that links PI3K/AKT/mTOR with MNK/eIF4E pathway. Thus, it has been demonstrated that a prolonged treatment of tumor cell lines or patients with rapamycin, an mTOR inhibitor, and its synthetic analogs (temsirolimus and everolimus) inhibits mTOR function but controversially, increases eIF4E phosphorylation and AKT activation, resulting in a mTOR-targeted therapy resistance through a compensatory feed-back mechanism between the AKT/mTOR pathway and MNK/eIF4E pathway [46,47,48,49].

MNK1 seems to play an important role in the interplay between both pathways (PI3K/AKT/mTOR and MNK/eIF4E) (Figure 2). Some studies suggest that MNKs have an additional role in post-transcriptional gene expression by controlling 7-methyl-guanosine cap-independent translation at internal ribosomal entry sites (IRES) located in the 5’-UTR (untranslated region) of some mRNAs in response to mTOR inhibitors. These inhibitors make tumor cell growth dependent on IRES-mediated cap-independent translation. Shi et al. demonstrated that MNK is a key regulator of rapamycin-induced IRES activity [50]. In other report, these authors demonstrated that MNK1, but not MNK2, regulates the IRES-dependent c-Myc translation in multiple myeloma (MM) cells during endoplasmic reticulum (ER) stress [51]. These authors propose that MNK1 stimulates the binding between two of the Myc ITAFs, hnRNP A1 and RPS25, during ER stress, facilitating ribosomal loading to the c-Myc IRES and leading to IRES-dependent translation. Brown et al. have shown that MNK promotes viral IRES dependent translation of polio/rhinovirus recombinant (PVSRIPO) [52], a fundamental requirement for the cytotoxic efficacy of this new oncolytic immunotherapy, currently in phase II clinical trials in adult patients with recurrent grade IV malignant glioma (NCT02986178). Authors propose that MNK, via the stimulation of mTORC1, exerts the inhibition of mTORC2 and AKT, which negatively regulates the Ser/Arg (SR)-rich protein kinase (SRPK) and its substrates, the SR-rich proteins, which are involved in mRNA splicing, export and translation, including viral IRES translation [52,53]. Recently, Brown and Gromeier have demonstrated that MNKs stimulate mTORC1 [54]. MNK binds to mTORC1, which promotes association with TELO 2 (PI3K related kinase (PIKK) stabilizer), and this interaction modulates mTORC1:substrate binding. It is interesting to point out that both mechanisms described above represent a point of convergence between PI3K/AKT/mTOR and MAPK/MNK pathways independent of eIF4E phosphorylation. 

**Figure 3:**
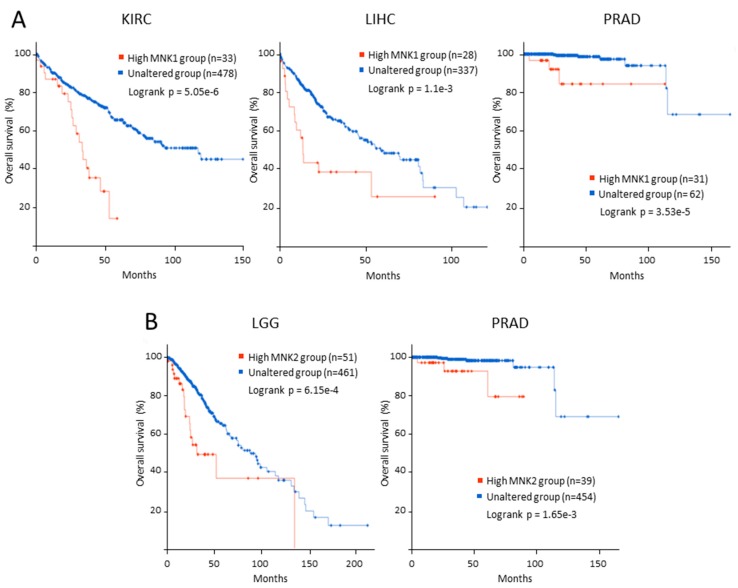
Survival analysis of MNKs overexpression in cancer patients from the TCGA database. (**A**) MNK1 overexpression significantly correlated with poor overall survival in KIRK, LIHC and PRAD patients, while (**B**) MNK2 overexpression was associated with worse overall survival in LGG and PRAD patients. Kaplan–Meier curves of overall survival were performed in 32 cancer types using the online tool cBioPortal based on mRNA expression data from the TCGA PanCancer Atlas dataset. High MNK expression (red line) is defined as the mRNA expression > 1.5 standard deviation above the mean. Log rank test *p* value < 0.05 was considered as statistical significance. Number of samples per group is note on plot. KIRC: Kidney renal clear cell carcinoma; LIHC: Liver hepatocellular carcinoma; LGG: brain lower grade glioma; PRAD: Prostate adenocarcinoma.

MNK isoforms are overexpressed in several types of cancer such as glioblastoma, lung, liver, ovarian and breast cancer [46,55,56,57,58,59] and their high expression levels are associated with worse prognosis [56,57,58,59]. To extend these results, we have performed Kaplan–Meier analysis on mRNA expression data from The Cancer Genome Atlas (TCGA) project (https://tcga-data.nci.nih.gov/tcga/). There is a significant correlation between MNK1 high expression and unfavorable overall survival in kidney, liver and prostate cancer and between MNK2 high expression and unfavorable overall survival in low grade glioma and prostate cancer patients (Figure 3). The importance of the overexpression of MNK1 or MNK2 in progression and survival in cancer could depend on the balance between both protein kinases in each tissue, as well as the ratio between the spliced isoforms a and b. Thus, Maimon et al. have found that the expression of MNK2a is decreased in breast, lung, and colon tumors, while MNK2b is correspondingly increased [60]. Interestingly, these authors reported that MNK2 *splice* variants have opposing roles in tumor development, MNK2a acts as a tumor suppressor while MNK2b has a pro-oncogenic role [60]. The antagonism between MNK2a and b could also occur for MNK1 isoforms. In triple-negative breast cancer, MNK1b overexpression was associated with shorter overall and disease-free survival times and its overexpression with gene transfection facilitates proliferation, migration, and invasion in breast cell lines [59].

Although previous studies were aimed at the use of eIF4E as a therapeutic target, the fact that this protein has a fundamental biological role in protein synthesis in normal cells is an obstacle to these strategies. Given that eIF4E and its phosphorylation are associated with processes linked to tumor progression and metastasis in a broad range of tumor types, and that MNKs are not essential [13], pharmacological inhibitors directed against MNK appear to provide an effective anti-tumor strategy non-detrimental for non-tumor cells. Furthermore, the combination of MNK and mTOR inhibitors increases anti-tumor response by inhibiting cell proliferation and inducing apoptosis compared to monotherapy, which has increased the studies driven to the use of combined therapies. We summarize the inhibitors against MNK1/2 described for cancer therapy (Table 1) and those clinical trials currently in progress with MNKs inhibitors (Table 2).

## 4. MNK in Hematological Cancers

Hematological malignancies as a whole occupy the third place in the global cancer classification, after lung and breast cancer. Among the several hematological cancer types, leukemias, lymphomas, and myelomas are the most frequent [113]. 

**Figure 4:**
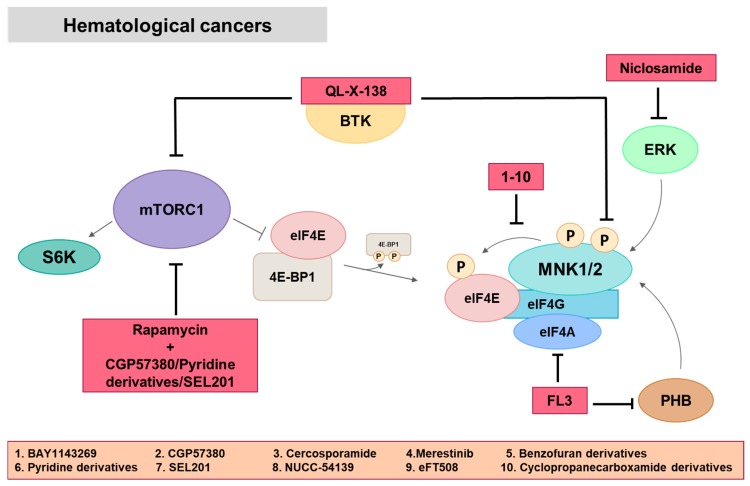
MNK in hematological cancers. Most of the MNK inhibitors act inhibiting eIF4E phosphorylation, although there are some exceptions. Niclosamide, an anthelminthic drug, affects eIF4E phosphorylation acting upstream ERK/MNK/eIF4E pathway. FL3 inhibits PHB affecting MNK/eIF4E and also eIF4A and therefore eIF4F complex formation. QL-X-138 targets BTK affecting PI3K/AKT/mTOR pathway and MNK1 phosphorylation. CGP57380, pyridine derivatives and SEL201 are also used in combination with rapamycin to enhance mTOR-targeted therapy. Red squares: inhibitors.

Acute myeloid leukemia (AML) is a genetically heterogeneous, malignant clonal disorder of the hematopoietic system that is characterized by uncontrolled proliferation of immature, abnormal blast cells and impaired production of normal blood cells [114]. In most of the published works, MNK has been demonstrated to be implicated in the pathogenesis of AML. MNK inhibition leads to a decrease in eIF4E phosphorylation levels, which entails antiproliferative effects, cell cycle arrest and an increase in cellular apoptosis mediated by high levels of cleaved PARP and decreasing MCL-1 (myeloid cell leukemia 1) levels. In addition, MNK inhibition has led to the design of new compounds such as MNKI-8e and 8i, pyrimidine analogs, MNKI-4, MNKI-57, merestinib, cercosporamide, BAY1143269, SEL201or NUCC-54139 [61,62,92,93,94,95,102,106,115,116,117,118] (Figure 4).

The World Health Organization (WHO) defines chronic myeloid leukemia (CML) as a chronic myeloproliferative neoplasm characterized by the presence of Philadelphia chromosome and the fusion oncogene *Bcr-Abl*. The inhibition of Bcr-Abl kinase by imatinib results in durable responses in early-stage CML patients, but less in late-stage disease, so patients can develop drug resistance. The joint inhibition of MNK and Bcr-Abl with the MNK inhibitor CGP57380 and with imatinib inhibits polysome assembly, decreasing proliferation and survival [119]. In patients who develop blast crisis (BC-CML), life expectancy is still less than 12 months [120]. The use of imatinib with MNK inhibitors prevents eIF4E phosphorylation in vivo with an antiproliferative effect that could help to combat late-stage disease and to understand other pathways and cellular processes that are dysregulated by Bcr-Abl [119]. In addition, pharmacologic targeting of MNK and mTORC1 kinases, employing rapamycin together with novel MNK inhibitors (MNK1/2 53–54 or MNKI-4 and MNKI-57) or niclosamide (an anthelminthic drug), abolished cell growth by triggering cell apoptotic death and abrogated eIF4E phosphorylation, which may offer a new therapeutic opportunity [76,96,110,121]. 

Acute lymphocytic leukemia (ALL) consists of the uncontrolled proliferation of an immature cell clone of lymphoid lineage (lymphoblasts) invading the bone marrow and infiltrates multiple organs and tissues. In this type of hematological cancer, it has been described that MNK1 overexpression and phosphorylation activates eIF4E, up-regulating downstream molecules such as MCL-1, c-Myc, survivin and the cyclin-dependent kinase (CDK) 2. MNK1 inhibition with CGP57380 prevents these events and can also overcome eIF4E activation induced by everolimus, sensitizing T-ALL cells to apoptosis [63].

On the other hand, pharmacological inhibition of the Bruton’s tyrosine kinase (BTK) is effective against a variety of B-cell malignancies. In 2016, a dual BTK/MNK inhibitor called QL-X-138 was developed with anti-proliferative effects in vitro and in patient-derived primary cells but for the chronic lymphocytic leukemia (CLL) treatment [108]. 

Diffuse large B cell lymphoma (DLBCL) is one of the most common types of lymphoma and accounts for approximately 30%–40% of non-Hodgkin lymphoma cases. It is a fast growing lymphoma with a high proliferation rate and aggressive behavior with a 30% of patients relapsing or refractory to first-line treatment [122]. Most of the MNK inhibitors used in this type of hematological cancer also block eIF4E phosphorylation, as it happens in others. Recently, Reich et al. have discovered an MNK inhibitor, eFT508 that blocked eIF4E phosphorylation and pro-inflammatory cytokine production without affecting proliferation in vitro and in vivo [107]. This inhibitor is being evaluated in a phase II clinical trial in lymphoma (Table 2). Likewise, Prohibitin (PHB) overexpression is associated with tumor aggressiveness. MNK inhibition by FL3, a synthetic flavagine and ligand of PHBs, determined antitumor activities in vitro and in vivo by inhibiting MNK-dependent eIF4E phosphorylation. This MNK1 inhibition reduced Bcl-2 and c-Myc expression, inducing apoptosis that would allow the treatment of rituximab resistant diseases [109]. 

Multiple myeloma (MM) is a malignant plasma cell disorder that is characterized by the presence of clonal plasma cell proliferation in bone marrow and over production of monoclonal paraprotein in the blood and/or urine [123]. In 2013, Mehrotra et al. established the regulatory role of MNK pathways as positive effectors in the generation of antineoplastic effects of type I IFNs in myeloproliferative neoplasms (MPNs) [97]. However, it remains to be elucidated whether other downstream effectors of MNK kinases apart from eIF4E are involved in the generation of IFN responses.

## 5. MNK in Solid Tumors

### 5.1. MNK in Breast Cancer

According to the WHO, breast cancer is the most frequent cancer among women, impacting 2.1 million women each year, and also causes the greatest number of cancer-related deaths among them. In 2018, it is estimated that 627,000 women died from breast cancer—that is approximately 15% of all cancer deaths among women [113]. Thus, the need for new therapies is revealed.

**Figure 5:**
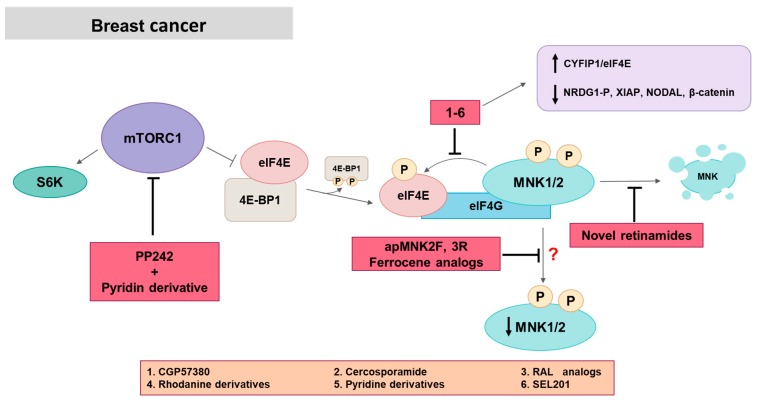
MNK in breast cancer. Most of the MNK inhibitors act inhibiting eIF4E phosphorylation, although there are some exceptions. Novel retinamides act through MNK degradation, and aptamers apMNK2F and 3R and Ferrocene analogs through an unknown mechanism independent of eIF4E phosphorylation. MNK inhibitors induce an increase in the association CYFIP1/eIF4E and the decrease of diverse pro-tumorigenic proteins. PP242, an mTOR inhibitor, and pyridine derivatives are also used in combination to enhance mTOR-targeted therapy. Red squares: inhibitors.

MNKs have a key role in breast cancer. MNK1a and MNK1b proteins and MNK1b mRNA levels are higher in breast tumors samples than in healthy tissues. In fact, MNK1b levels are significantly increased in triple-negative breast cancer tumors (TNBC) and are associated with poorer overall and disease-free survival [59]. MNK inhibition resulted in reduced proliferation and cell viability, supported by the downregulation of cyclin D1 and cell cycle arrest in MDA-MB-231 cells in response to resorcylic acid lactone (RAL) analogs, CGP57380, rhodanine analogs or pyridine derivatives and/or to a combination of MNK and PI3K inhibitors [55,98,124,125,126]. However, the use of different inhibitors reveals other processes affected by MNK blockade. VNLG-152, a retinamide derivative, and its racemic form VNLG-152R degraded MNK1 and blocked eIF4E phosphorylation producing a decrease in colony formation, migration and invasion, inducing cell death by apoptosis and affecting the cell cycle in MDA-MB-231 and MDA-MB-468, and suppressed the growth of MDA-MB-231 tumor xenografts and in TNBC patient-derived xenograft (PDX) model [84,85,86]. Compound MNK-7g is able to inhibit eIF4E phosphorylation and to block the migration of MDA-MB-231 without affecting proliferation [99]. However, eIF4E involvement does not always occur after MNK inhibition, so the effect of the different inhibitors on the kinase could affect or not affect eIF4E. Aptamers (ssDNA molecules that adopt tertiary structures) apMNK2F and apMNK3R, which inhibit translation and decrease cell viability, migration, and colony formation in MDA-MB-231 cells [112], and Ferrocene analogs, MNK inhibitors that inhibit cell viability and spheroid growth [127], do not produce any effect on eIF4E phosphorylation. Thus, the study of possible MNK substrates different from eIF4E is still ongoing. A group of researchers identified MNK1 as a YB-1 target, which is overexpressed in trastuzumab-resistant cell lines and that the role of MNK1 would be independent of eIF4E phosphorylation. They demonstrated that MNK1 levels are regulated by phospho-YB-1, which is downstream from RSK signaling and, also, RSK directly phosphorylates MNK1. Therefore, inhibiting YB-1 function increased sensitivity to trastuzumab by reducing the expression of MNK1, EGFR, MET, CD44 and anti-apoptotic proteins such as MCL-1, cIAP1 and cIAP2 [128]. In another study, a novel mechanism by which MNKs could control the expression of specific protein has been proposed, showing that MNK inhibition increased the association of CYFIP1 with eIF4E. MNKI-1 inhibited eIF4E phosphorylation and migration of mouse embryonic fibroblasts (MEFs), MDA-MB-231 and SCC25 cells with little or no effect on cell viability or proliferation [100]. Using the same inhibitor, Tian et al. demonstrated that MNK1 inhibition decreased the phosphorylation of the metastasis suppressor NDRG1 in MDA-MB-231 cells [64] (Figure 5). 

XIAP protein is an apoptosis inhibitor that is overexpressed in high-grade breast cancer and in inflammatory breast cancer (IBC) patient tumors. XIAP is necessary for the constitutive activation of the NFkB pathway in IBC and the XIAP-NFkB axis directly correlates with the tumor growth rate in vivo. Interruption of MNK signaling led to a reduction in XIAP expression. XIAP mRNA contains IRES and MNK regulation may function to facilitate XIAP translation in IBC. Thus, XIAP acted as a link between MAPK and NFkB signaling to control IBC proliferation and tumor aggression [129].

MNK1/NODAL has been identified as a key signaling axis regulating the progression and breast cancer recurrence as metastatic disease. MNK1 controlled NODAL protein levels, possibly on the level of mRNA translation. The data showed a positive correlation between MNK1 activity and the expression of NODAL and vimentin, regulators of invasion and metastasis. MNK1 inhibition with SEL201 could block NODAL signaling to suppress disease [103]. However, all the possible downstream factors and MNK-interactions are yet to be discovered.

The activation of the MNK/eIF4E/β-catenin axis is involved in breast cancer cell response to chemotherapy. A study has proposed *β-catenin* as a new eIF4E-targeted tumor promoting genelike *MCL-1* and *cyclin D*. These authors showed that CGP57380 and cercosporamide prevent chemotherapy-induced eIF4E phosphorylation and β-catenin activation, inhibiting proliferation and inducing apoptosis of breast cancer cells in vitro and in vivo [65]. 

### 5.2. MNK in Lung Cancer

Lung cancer is the most common cancer and the world-leading cause of cancer-related death [113]. Histologically, lung cancer is divided into two main types: small-cell lung cancer (SCLC) and non-small cell lung cancer (NSCLC). SCLC is not very common (15% of all lung cancer cases) but is more metastatic and aggressive than NSCLC. NSCLC is less aggressive than SCLC but more frequent (85% of all cases) and it can be divided into three subtypes: adenocarcinoma (50%), squamous cell carcinoma (30%) and large cell carcinoma (10%) [130]. 

The active form of MNK1, p-MNK1 (Thr197/202), and phosphorylated eIF4E, p-eIF4E, are increased in lung cancer and correlate with poor overall survival of NSCLC patients [66,131]. In addition, high levels of p-MNK1 might act as an independent poor prognostic biomarker for these patients [66]. On the other hand, MNK2 is overexpressed in NSCLC promoting cell proliferation, migration and invasion in vitro and in vivo through 4E-BP1/eIF4E and ERK/eIF4E pathways. High expression of MNK2 correlates with lymph node metastasis and poor overall survival rates in patients with NSCLC [56]. The isoform MNK2a is a tumor suppressor mechanism that is lost in some lung tumors [60]. All these data reflect that targeting MNK-proteins might be a potential therapeutic strategy for treatment in NSCLC patients. 

**Figure 6:**
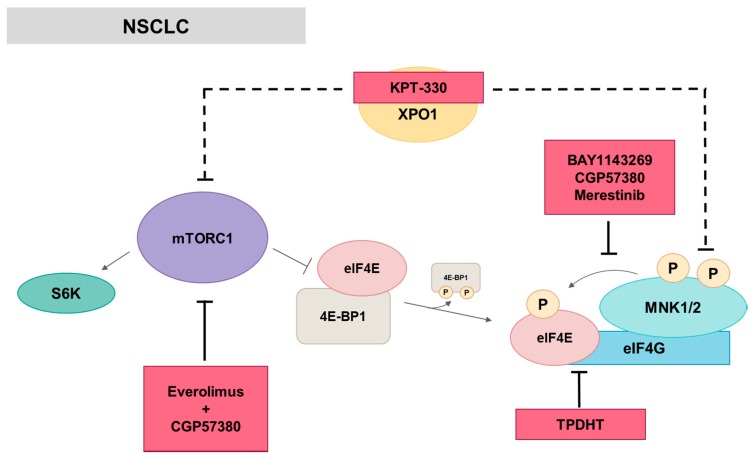
MNK in NSCLC. Most of the inhibitors act inhibiting eIF4E phosphorylation, whereas TPDHT disrupts eIF4E/eIF4G interaction and KPT-330 targets exportin 1 (XPO1) downregulating mTOR and MNK1 phosphorylation among others. CGP57380 is also used in combination with Everolimus to enhance mTOR-targeted therapy. Red squares: inhibitors.

Some MNK inhibitors have been developed in the last few years with satisfactory results such as BAY 1143269 [61], a potent MNK1 inhibitor identified by high-throughput screening. BAY 1143269 is more potent than CGP57380 and cercosporamide, the classical MNK1/MNK2 inhibitors [67,77]. Treatment with BAY 1143269 inhibits eIF4E phosphorylation and leads to cell cycle deregulation in NSCLC cell lines through a G0/G1 arrest and a reduced expression of several cell cycle factors, including different cyclins. Treating NSCLC cell lines with BAY 1143269 also decreases its migratory potential, induces apoptosis and causes a reduction in several key factors in the epithelial-mesenchymal transition (EMT). In addition, this MNK1 inhibitor shows anti-cancer activity as monotherapy in different NSCLC cell lines and PDX models. Combinational therapy with chemotherapeutics such as docetaxel significantly improves anticancer activity compared to monotherapy in vivo *(*Figure 6). BAY 1143269 started to be evaluated in clinical trials as a combinational therapy with docetaxel in NSCLC in 2015 (Table 2). Moreover, merestinib (LY2801653), a multi-kinase inhibitor with activity against MNKs among other protein kinases, inhibits tumor growth and metastasis in NSCLC models [78,79,80], and is currently used in clinical trials (phase II)—not only in NSCLC, but also in AML, biliary tract and colorectal cancer (Table 2).

Targeting different proteins than MNKs, both mTORC1/4E-BP1 and MNK1/eIF4E axis are inactivated in NSCLC cell lines as occurs with exportin 1 (XPO1) inhibitor KPT-330, which disrupts the eIF4F translation initiation complex due to a downregulation of mTOR and MNK1 and phosphorylation of mTOR, p70S6K, 4E-BP1, MNK1 and eIF4E [132]. Moreover, TPDHT is a bromophenol-thiazolylhydrazone hybrid that inhibits proliferation in a variety of tumor cells, especially in the human lung cancer cell A549, in which also induces apoptosis, G0/G1 phase arrest and antitumor activity in vivo. TPDHT targets eIF4E disrupting the interaction of eIF4E/eIF4G through ERK/MNK/eIF4E pathway [133].

On the other hand, PI3K/AKT/mTOR pathway is frequently activated in NSCLC and involved in lung tumorigenesis [134,135]. Currently, several inhibitors of the PI3K pathway have been developed and are undergoing evaluation in preclinical and clinical studies (reviewed in [136]), specially mTOR inhibitors such as rapamycin and its analogs (rapalogs). However, the prolonged treatment of human lung cancer cells with rapalogs results in an mTOR-targeted therapy resistance through the compensatory feed-back mechanism between AKT/mTOR pathway and MNK/eIF4E pathway [48,49]. Therefore, combination therapy with both mTOR and MNK inhibitors might be an effective therapeutic strategy to enhance mTOR-targeted cancer therapy in NSCLC. Thus, Wen et al. showed that the MNK inhibitor CGP57380 abrogates the eIF4E phosphorylation induced by the mTOR inhibitor RAD001 (everolimus) in NSCLC cells and the combination of both inhibitors exerts synergistic inhibitory effects on cell proliferation, colony formation and inhibits tumor growth of lung cancer xenografts [66]. In addition, apoptosis is induced by decreasing several anti-apoptotic factors, including MCL-1, whose expression is remarkably increased and correlated with poor prognosis in NSCLC patients and is reduced after CGP57380 treatment in lung cancer cells [68]. Thus, MCL-1 might act as a novel biomarker in these patients.

### 5.3. MNK in Prostate Cancer

Prostate cancer (PCa) is the second most frequent cancer in males and the fifth leading cause of cancer-related death in this gender [113]. Although there is no direct evidence for MNKs overexpression in prostate cancer patient samples, a few studies have shown that elevated eIF4E levels and hyperphosphorylation, which reflect high MNKs activity, were correlated with disease progression [137,138].

**Figure 7:**
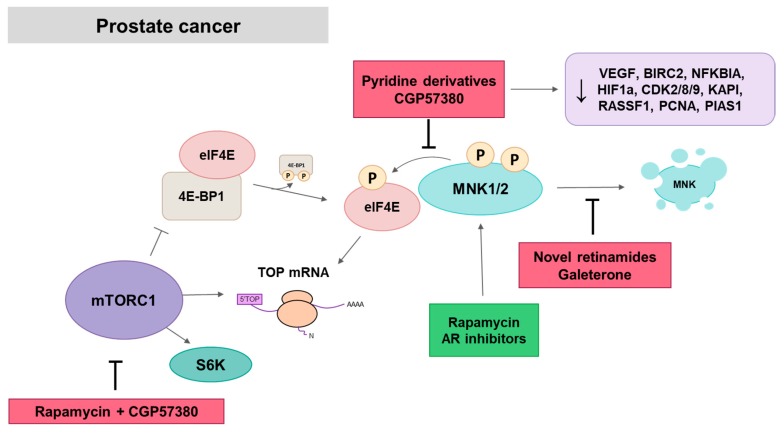
MNK in prostate cancer. Novel retinamides and galeterone and its analogs promote MNK proteasomal degradation, whereas CGP57380 and pyridine derivatives inhibit eIF4E phosphorylation. Inhibition of MNK by CGP5738 decreases translation of diverse pro-tumorigenic proteins and TOP mRNAs, which is further reduced by rapamycin concomitant treatment. mTOR and AR inhibitors increase MNK activity as a resistance mechanism. Red squares: inhibitors; Green square, activators.

MNK/eIF4E pathway involvement in prostate tumor progression has been also reported in vitro and in animal models. Furic et al. have shown that knock-in mouse expressing a non-phosphorylable form of eIF4E was resistant to prostate tumor development in a mouse model based on loss PTEN function [137]. Polysome-profiling of MEFs isolated from knock-in mouse and PCa cell lines treated with CGP57380 allowed the identification of MNK/eIF4E-dependent mRNAs as VEGFC, BIRC2, MMP3, and NFKBIA, pro-tumorigenic proteins whose decrease was associated with tumor development resistance. Microarray analysis of polysome-associated mRNAs of a different PCa cell line using CGP57380 has demonstrate that MNKs are required for translation of mRNAs involved in hypoxia response (specifically HIF1α transcription factor), and cell cycle progression (CDK2, CDK8, CDK9, KAP1, RASSF1, PCNA and PIAS1), which may explain the antiproliferative effect of MNK inhibition in vitro [47] (Figure 7). MNK1 silencing also caused a decrease in viability of PCa cells [87].

The androgen receptor (AR) is a main driver of PCa development and progression. Initially, patients are treated with androgen-deprivation therapy, but PCa often progresses to a castration-resistant prostate cancer (CRPC), with worse prognosis. D’Abronzo et al. have shown that AR inhibition led to an increase in eIF4E phosphorylation and cap-dependent translation that confers resistance to AR antagonists as bicalutamide [139]. Moreover, CGP57380 or eIF4E depletion sensitizes CRPC to anti-androgen therapies. Since AR plays a pivotal role in prostate cancer and MNK/eIF4E pathway is involved in cancer resistance and progression, dual AR/MNK inhibition seems to be a promising PCa therapy. Two families of dual AR/MNKs inhibitors have been proven to be effective in PCa, novel retinamides and galeterone analogs.

Novel retinamides are retinoic acid metabolism blocking agents that promote the ubiquitin-mediated proteasomal degradation of AR and MNKs as an off-target effect [87]. Some novel retinamides like VNLG-152, VNHM-1-81, VNHM-1-66, and VNHM-1-73, suppress growth, migration, and invasion in a variety of PCa cell lines, and reduce tumor growth and EMT in CRPC mice xenografts [85,87,88]. 

Galeterone and galeterone analogs are AR antagonists/degradation inducers and CYP17 inhibitors that lead to the ubiquitin-proteasomal degradation of MNKs and consequently decrease eIF4E phosphorylation. Galeterone and its analog VNPT55 inhibit PCa cells migration and invasion through the downregulation of EMT markers such as Snail, Slug, N-Cadherin, vimentin and MMP-2/-9 and up-regulation of E-cadherin [89]. The Next generation Galeterone analogs, VNPP414, and VNPP433-3b, induce apoptosis, inhibit proliferation, migration, and invasion by modulating EMT and stem cell markers in PCa cells and suppress tumor growth in a CRPC xenograft mouse model. Furthermore, gal and NGGAs are effective in enzalutamide, docetaxel and mitoxantrone-resistant PCa cells and have a synergistic effect with docetaxel and enzalutamide [90]. Many other MNKs inhibitors have shown promising antitumor effects in prostate cancer models such as 3-azido withaferin A [140], a dual modulator of Ras/MNK and PI3K/AKT/mTOR pathways, 2′H-spiro[cyclohexane-1,3′-imidazo[1 ,5-a] pyridine]-1′,5′-dione derivatives [98] and eFT508, currently in phase II trials for patients with advanced CRPC (Table 2). 

Bianchini et al. have demonstrated that the compensatory feedback between PI3K/AKT/mTOR and RAS/MAPK/MNK pathways occurs in prostate carcinomas and preserves tumor progression [47]. PCa cell lines with low AKT/mTOR activity have high levels of eIF4E phosphorylation and a stronger antiproliferative response to MNK inhibition than to rapamycin, while PCa cells with mutated PTEN and constitutively activated AKT/mTOR pathway have lower eIF4E phosphorylation levels that can be robustly induced with mTOR inhibition and counteracted by co-treatment with CGP57380. In CRPC, resistance to rapamycin was also associated with upregulated eIF4E phosphorylation induced by AR inhibition [139]. In PCa cells, rapamycin-induced eIF4E phosphorylation is mediated by an increase in MNK2-activity dependent on phosphorylation at Ser437 [141]. MNK inhibition alone reduced polysomal recruitment of terminal oligopyrimidine messenger RNAs (TOP) mRNAs, which are mRNAs with a common sequence at the 5′ that encodes ribosomal proteins and components of translational complex. The translation of these mRNAs is mainly regulated by mTORC1 activity in response to growth factors. Concomitant treatment with CGP57380 and rapamycin has additive effects in reducing polysomal recruitment of TOP mRNAs. This result suggests the additional translation control of TOP mRNAs by the MNK/eIF4E pathway. Moreover, simultaneous mTOR and MNK inhibition suppress protein synthesis, cell proliferation and cell cycle, with a decrease in cyclin D1, cyclin A and cyclin B [47].

### 5.4. MNK in Gastrointestinal Cancer

Gastrointestinal cancer includes malignant conditions of the gastrointestinal tract and other organs involved in digestion, including the esophagus, stomach, biliary system, pancreas, small intestine, large intestine, rectum and anus (colorectal tract). Altogether, these tumors are responsible for more death from cancer than other body systems.

**Figure 8:**
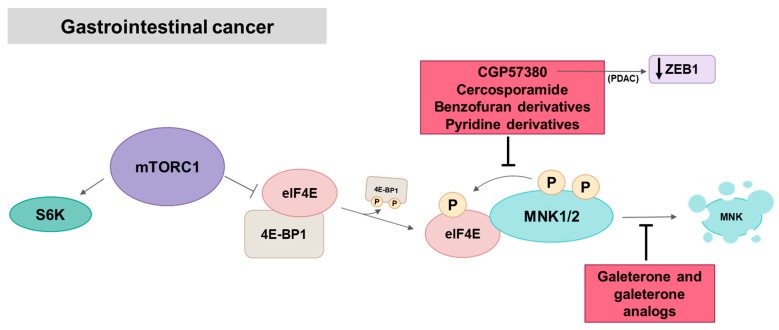
MNK in gastrointestinal cancer. Most of the inhibitors act inhibiting eIF4E phosphorylation. Galeterone and its analogs promote MNK proteasomal degradation. In PDAC cells, the MNK inhibitor CGP57380 induces a decrease in the levels of the transcription factor ZEB1. Red squares: inhibitors.

Pancreatic ductal adenocarcinoma (PDAC) is the most common type of pancreatic cancer and is the seventh leading cause of cancer death, with one of the highest mortality/incidence ratios between solid tumors [113]. The advanced stage at diagnostic and limit or ineffective chemo and radio-therapies contribute to poor outcome. Gemcitabine, one of the first-line chemotherapies used in PDAC, has limited efficacy due to the development of chemoresistance, partially associated with the dysregulation of mRNA translation. Adesso et al. showed that gemcitabine upregulates the oncogenic splicing factor SRSF1 and promotes splicing of the MNK2 increasing MNK2b isoform, which confers drug resistance by increasing eIF4E phosphorylation [38]. In accordance, MNK inhibition by CGP57380, and MNK2 or SRSF1 silencing synergistically enhance the anti-tumor effect of gemcitabine by promoting apoptosis. Moreover, MNK inhibition increases the cytostatic effect of cisplatin and rapamycin in PDAC cells. MNKs may be involved in PDAC pathogenesis and progression, as MNK1 is highly expressed in pancreatic acinar cells in mice and is activated upon induction of pancreatitis, a major risk factor for the development of PDAC [69]. Furthermore, increased eIF4E phosphorylation is associated with a higher tumor grade, early disease onset and worse prognosis [38]. MNKs also play an important role in regulating EMT in PDAC cells. Pharmacological inhibition and genetic depletion of MNKs, mainly MNK2, reduces protein expression of the EMT-activating transcription factor ZEB1, leading to the reversion of EMT and decrease in migration and invasion of PDAC cells. In addition, MNK inhibition decreases cell growth and reverses EMT increasing E-cadherin mRNA levels and decreasing vimentin mRNA levels in human PDAC organoids. Kumar et al. also showed that 5-fluorouracil-chemoresistant PDAC cells, which have undergone EMT and have increased ZEB1 levels, are sensitive to MNK inhibition and have fewer cancer stem cells after CGP57380 treatment [70]. Kwegyr-Afful et al. demonstrated that galeterone and its analogs (VNPT55, VNPP414, and VNPP433-3β) synergize with gemcitabine and inhibit PDAC migration, invasion and proliferation of gemcitabine-naïve and resistant PDAC cells through downregulation of MNK1/2 and suppress tumor growth of PDAC xenografts in mice [91] (Figure 8). Based on these results, galeterone is currently in clinical trials (phase II) in advanced PDAC alone and in combination with gemcitabine (Table 2).

Colorectal Cancer (CRC) is the fourth diagnosed cancer and the second cause of cancer death in the world [113]. There are some MNK1/2 inhibitors tested in CRC, such as cercosporamide, which suppresses eIF4E phosphorylation in colon cancer cell lines blocking the growth of HCT116 colon carcinoma xenograft tumors [77]; 6-hydroxy-4-methoxy-3-methylbenzofuran-7-carboxamide derivatives compounds 5o and 8k, that also exhibit anti-proliferative activity and block eIF4E phosphorylation in the CRC HCT-116 cell line [82]; 42i, a pyridine-aminal derivative synthesized as MNK1/2 inhibitor that significantly blocks eIF4E phosphorylation in colon cancer CT-26 cell line and inhibits tumor growth in CT-26 allograft model [83] and 4t, a pyridine derivative with anti-proliferative activity against CRC cell lines among others [98]. Targeting different proteins than MNKs, two compounds are found to downregulate MNK1 in CRC, CDKI-73 [142] and metformin [143]. Regarding MNK inhibitors in clinical trials, the MNK1/2 inhibitor eFT508 is being evaluated in phase II in colorectal cancer patients alone or in combination with Avelumab. Merestinib is being evaluated in phase I in combination with Ramucirumab in colorectal cancer patients (Table 2).

High expression of MNK1 is more frequent in hepatocellular carcinoma (HCC) tissues than in normal liver tissues, which correlates with poor overall survival. This MNK1 overexpression enhances proliferation, migration and invasion in HCC cell lines [58]. Cercosporamide blocks eIF4E phosphorylation inhibiting proliferation and angiogenesis in HCC cell lines and, in combination with cisplatin, results in greater efficacy than a drug alone in vitro and in vivo [144]. Regarding MNK-inhibitors in clinical trials, eFT508 was evaluated in a phase II trial in hepatocellular carcinoma patients. Merestinib is being evaluated in phase I in combination with cisplatin and gemcitabine in cholangiocarcinoma and biliary tract carcinoma patients, among others (Table 2).

### 5.5. MNK in Brain and CNS Tumors

Gliomas are the most common primary brain tumors in adults and arise from the glial tissue. Based on histological criteria, WHO has classified diffuse gliomas into lower-grade astrocytomas or oligodendrogliomas and high-grade astrocytomas, also known as glioblastoma multiforme (GBM), the most prevalent and aggressive type of brain cancer [145]. Clinical studies have demonstrated that there is a higher expression of MNK1 at protein levels in GBM tumor samples and glioma cell lines compared with non-tumorous brain tissue and normal human astrocytes, respectively. Microarray analysis also revealed upregulated MNK1 transcript levels in GBM and lower grade astrocytomas, without changes in MNK2 expression [46]. Moreover, there are higher levels of p-MNK1 and its substrate p-eIF4E in astrocytoma tissues compared to normal brain tissues, which were associated with tumor recurrence. Furthermore, p-MKN1 levels were positively correlated with p-eIF4E levels, which in turn are associated with the grade of astrocytomas, tumor size, and unfavorable prognosis, and inversely correlated with overall survival rates [146]. Across GBM subtypes, both *MKNK1 and MKNK2* genes are highly expressed in mesenchymal subtype GBM, while only the *MKNK1* expression correlates with the mesenchymal glioma stem cells marker CD44 and predicts poor survival in GBM when both genes are upregulated [81,147]. Several studies have shown an oncogenic role for MNK1 and MNK2 in glioma development. MNK1 knockdown as well as inhibition by CGP57380 decrease in vitro and in vivo oncogenic activity along with a significant reduction in eIF4E phosphorylation in human glioma cell lines [40,46].

Primary central nervous system lymphoma (PCNSL) is an uncommon subtype of extranodal non-Hodgkin’s lymphoma that arises inside the central nervous system. Muta et al. reported an overexpression of both phosphorylated and unphosphorylated eIF4E in samples from PCNSL patients and demonstrated the contribution of MNK1/eIF4E pathway in tumoral growth of brain malignant lymphoma cells in vitro and in a mouse xenograft model using CGP57380 [71].

**Figure 9:**
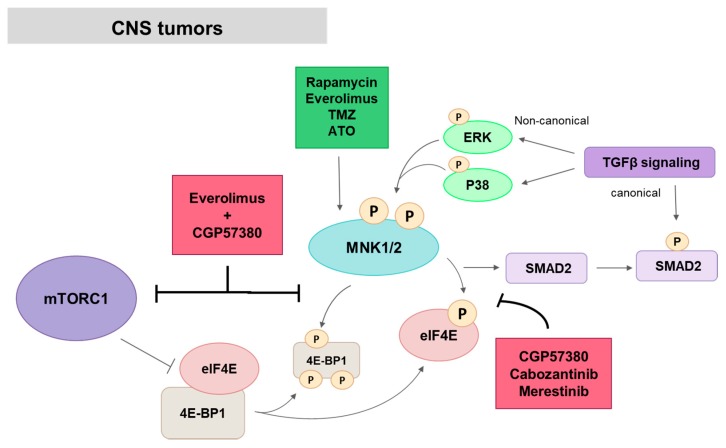
MNK in brain and CNS tumors. CGP57380, cabozantinib and merestinib act inhibiting eIF4E phosphorylation. MNK1 knockdown or inhibition by CGP57380 reduces SMAD2 expression and consequently TGFβ canonical signaling. MNK activity is increased by TGBβ non-canonical signaling and by antitumor glioma therapies as TMZ, ATO or mTOR inhibitors. CGP57380 in combination with everolimus inhibits MNK1-dependent phosphorylation of 4EBP1 and enhance mTOR-targeted therapy. Red squares: inhibitors; Green square, activators.

MNKs might regulate a specific set of genes depending on the cancer type or the particular signaling triggered by different therapies. Some specific MNK1 targets have been described in glioma. Microarray polysome-associated RNAs analysis in MNK1-depleted BS125 GBM cell line revealed that MNK1 regulates the translation of proteins involved in TGFβ (Transforming growth factor β) signaling. In particular, SMAD2, one of the main TGFβ signal transducers was found to be decreased after MNK1 knockdown or inhibition by CGP57380 and had a positive correlation with MNK1 expression in GBM samples. SMAD2 protein synthesis inhibition led to a decreased vimentin expression that could be associated with MNK1 control of cellular motility [46]. There is another point of convergence between TGFβ and MNK1 pathways. Several studies have described the activation of MNK1 by increasing phosphorylation of upstream p38 and ERK1/2 kinases after TGFβ treatment [46,148], which facilitates the translation of pro-metastasis mRNAs such as snail and MMP3 [149]. In PCNSL, treatment with CGP57380 reduced cyclin D1 expression, which was associated with lower proliferation rates [71] (Figure 9).

As stated above, targeting mTOR and MNK simultaneously may increase efficacy against cancer. Thus, combination of CGP57380 and rapamycin or RAD001 (everolimus) has an additive antiproliferative effect in glioma cell lines and reduces tumor growth in an orthotopic GBM mouse model [46,72]. In the same way, depletion of MNK1 by specific siRNAs enhanced sensitivity to rapamycin, while MNK1 overexpression reduced its inhibitory effects, with a positive correlation between MNK1 protein levels and rapamycin resistance [46]. Concomitant treatment of RAD001 with CGP57380 or MNK1 knockdown abrogates RAD001-induced eIF4E phosphorylation and additionally inhibits MNK1-dependent phosphorylation of 4EBP1 at serine 65. Consequently, the binding ratio of 4EBP1-eIF4E is increased, which results in a markedly decrease in global protein translation that could be related to the additive growth-inhibitory effects [46,72].

Medulloblastoma is an embryonal tumor of the cerebellum among the most frequent malignant childhood brain tumors [150]. In medulloblastoma cell lines, rapamycin-induced MNK2-mediated eIF4E phosphorylation enhanced antineoplastic effect, and is independent of MAPKs canonical MNK-activating pathway [73].

Additional studies had reported MNK-mediated altered regulation of translation initiation as a resistance mechanism to other antitumor glioma therapies as Temozolamide (TMZ) [151], arsenic trioxide (ATO) [147] or ionizing radiation [152], all of them related with increments in cap-dependent translation. TMZ, a chemotherapeutic DNA-damaging drug currently used in the standard treatment of GBM, increases eIF4E phosphorylation in glioma cells. ERK and MNK inhibitors, as well as MNK1 specific depletion, inhibit TMZ-induced eIF4E phosphorylation and enhanced TMZ antiproliferative effects, suggesting a key role of ERK/MNK1/eIF4E in TMZ resistance. Quantitative phosphoproteomics analysis in the presence of TMZ has shown that MNK affects phosphorylation status of proteins involved in the cellular response to stress and DNA damage and has allowed to identify MNK-dependent eIF4G1 phosphorylation sites, which are necessary for eIF4E phosphorylation and TMZ resistance mechanism of glioma cells [151]. ATO, an FDA-approved drug for leukemia, is currently under phase I/II clinical trial in GBM. In GBM cells, ATO increases eIF4E phosphorylation and translation of anti-apoptotic mRNAs by directly binding and activation of MNK1. In addition, resistance to ATO was positively correlated with eIF4E phosphorylation in an intracranial GBM PDX model, while patients from an ATO clinical trial with lower ATO response had higher MNK activity. CGP57380 and MNK1 silencing prevent ATO-induced eIF4E phosphorylation and increase ATO antiproliferative effect, which suggests the MNK1/eIF4E pathway as a resistance mechanism to ATO [147]. These results suggest an attractive approach to overcoming resistance mechanisms or sensitizing cancer to chemotherapy, by targeting MNKs activity in a combination of drug therapies.

Some studies evidenced the synergistic effect of MNK inhibition and other targeted therapies in central nervous system tumors. In malignant peripheral nerve sheath tumors (MPNSTs), a rare and aggressive sarcoma subtype of neural origin, Lock et al. have demonstrated high MNK/eIF4E activity in primary human tumors and an enhanced antineoplastic effect of MEK inhibitor PD901 combined with MNKs knockdown or inhibition in vitro and in vivo in a mechanism dependent of eIF4E phosphorylation levels [111]. Furthermore, MNK inhibition has additive antitumor effects in combination with alpelisib, a PI3Ka inhibitor, in medulloblastoma mouse xenograft models [153].

Merestinib inhibits tumor growth and has anti-angiogenic and anti-proliferative effects in a subcutaneous and intracranial GBM xenograft model [78,81]. Antitumor effects of merestinib in GBM lines and patient-derived mesenchymal glioma stem cell lines are related to the inhibition of eIF4E phosphorylation and a decrease in global protein synthesis and cyclin D1/D2 translation [81]. Cabozantinib, another MET/multikinase inhibitor with MNKs as direct targets, exhibited anti-tumor activity in vitro and in a genetically engineered mouse MPNST model by suppressing eIF4E phosphorylation [111].

### 5.6. MNK in Other Solid Tumors

MNKs activity in thyroid cancer has been mainly associated with drug resistance mechanisms. In thyroid cancer cell lines, an increase in MNK-dependent eIF4E phosphorylation is observed after DNA damage by cisplatin [154] or by radionuclide therapy with radiolabeled gastrin analogs [151] and after inhibition of BET proteins, which function as transcriptional co-activators of oncogenic genes [155]. MNK inhibition or knockdown blocks the increased eIF4E phosphorylation and enhances the antitumor effects of these therapies in vitro [151] and in vivo [154,155]. In addition, MNK inhibition or genetic depletion alone inhibits proliferation and induces apoptosis of anaplastic thyroid cancer cells, the most aggressive type of thyroid cancer, and reduces tumor growth in a mouse xenograft model [154].

In skin cancer, melanoma is the most fatal due to its metastatic and aggressive characteristics. MNK1 activity is associated with invasion and metastasis in different types of melanoma, including KIT-mutant [104] and BRAFv6000Emutant melanoma [105]. The MNK1/2 inhibitor SEL201 inhibits invasion in both melanoma cells and in vivo melanoma models [102,104,105]. Yang et al. have demonstrated that MNK1 regulates melanoma metastasis through the upregulation of the angiopoietin-like 4 (ANGPTL4) protein, a regulator of MMPs, thus enabling the subsequent expression of MMPs that promote melanoma cell invasion [105].

MNKs are also involved in gynecologic cancers. Thus, higher levels of MNK1 in epithelial ovarian cancer indicate poorer clinical outcomes. In addition, MNK1 knockdown and inhibition decreased ovarian cancer cell viability [57]. Liu et al. demonstrated that MNK1 is involved in the resistance of ovarian cancer cells to chemotherapy [156]. They observed increased phosphorylation levels of ERK, MNK1, and eIF4E in ovarian cancer cells exposed to chemotherapy, as well as in ovarian cancer patients. p-eIF4E overexpression resulted in resistance, whereas eIF4E depletion sensitized ovarian cancer cells. In cervical cancer, MNK regulates Wnt/β-catenin pathway. Thus, activated MNK/eIF4E induced the activation of Wnt/β-catenin in cancer, but not in normal cervical cells, through an increase in eIF4E overexpression and phosphorylation, which promoted growth and migration. In parallel, MNK inhibition prevented eIF4E-mediated Wnt/β-catenin activation, leading to decrease cervical cancer growth, migration, and survival. The combination of CGP57380 and Paclitaxel achieved greater activity than a single drug alone [101].

There is an overexpression of p-eIF4E and p-MNK1 in nasopharyngeal carcinoma (NPC) compared to non-cancerous nasopharyngeal epithelial tissues, which is associated with lymph node metastasis and poor survival [157,158]. In NPC, β-catenin signaling is aberrantly activated which is a factor of poor prognosis. Furthermore, p-eIF4E has been reported to activate the Wnt/β-catenin pathway. CGP57380 decreases proliferation, colony formation, migration and invasion in NPC cell lines and in vivo through the downregulation of β-catenin in the nucleus. Accumulation of β-catenin in the cytoplasm enhances intercellular adhesions and reduces the expression of EMT markers such as vimentin, N-cadherin and slug [74]. Pyrimidine derivatives have also acquired relevance in NPC, as 12j, the MNK1 inhibitor that inhibits kinase activity and cell proliferation and induces apoptosis in NPC and renal cell carcinoma (RCC) [75]. In clear cell RCC, one of the most common neoplasms of the kidney, high levels of p-eIF4E are associated with a longer recurrence-free interval after nephrectomy. In human RCC cell lines, eIF4E phosphorylation is mainly dependent on MNK2a isoform and its inhibition with CGP57380 or specific MNK2a genetic depletion enhanced migration and invasion and vimentin expression, which suggest that MNK2a may function as a metastasis suppressor [159].

## 6. Conclusions

MNKs proteins are key actors in tumor progression and metastasis in many human tumors playing an important role controlling the expression of specific proteins involved in cell cycle, cell survival and cell motility. In the last years, new key proteins implicated in tumor biology have been included among those directly regulated by MNKs. Thus, MNKs regulate translation of proteins involved in cell growth such as c-Myc, cyclin D1, β-catenin or CDK2, in antiapoptotic processes such as MCL-1, XIAP and survivin or in metastatic processes, migration, invasion, and EMT, like NODAL, SMAD2, NDRG1, ANGPTL4 or ZEB1. The study of the exact mechanism by which MNKs cause a tumorigenic effect in the different cancer types has been highly relevant to consider these proteins as potential therapeutic targets. In fact, it has been shown that, in addition to the phosphorylation of eIF4E, MNKs are capable of producing their effect through other substrates such as hnRNP A1, PSF or Sprouty 2 (Figure 2).

Moreover, MNKs seem to play an important role in the interplay between the Ras/MNK and PI3K/AKT/mTOR pathways, two critical signaling pathways involved in tumorigenesis and chemoresistance that are frequently deregulated in a broad variety of cancers.

From these results, regulating the expression or activity of MNKs has been a therapeutic strategy that has acquired enormous relevance. For this reason, in recent years there have been many investigations aimed at developing MNK inhibitor molecules that allow neutralizing the tumorigenic effect of these proteins. Inhibitors developed recently, some of which are already in different phases of clinical trials, open a window of hope for the pharmacological treatment targeting MNKs, in monotherapy or in combined therapy, of many tumors.

## Figures and Tables

**Table 1:** MNK inhibitors described for cancer therapy.

Compound	Comments	Indications	Reference
BAY1143269	MNK inhibitor	NSCLC, leukemia	[61]
CGP57380	MNK inhibitor	NSCLC, breast cancer, leukemia, lymphoma, myeloma, glioma, PCNSL, medulloblastoma, nasopharyngeal carcinoma, PDAC, ovarian cancer	[40,46,50,51,55,62,63,64,65,66,67,68,69,70,71,72,73,74,75]
Cercosporamide	MNK inhibitor	NSCLC, colorectal cancer, liver cancer, breast cancer, leukemia, lymphoma	[40,62,65,76,77]
Merestinib	Multi-kinase inhibitor	NSCLC, leukemia, glioma	[78,79,80,81]
Benzofuran derivatives (5o,8k)	MNK inhibitors	Colorectal cancer, leukemia	[82,83]
Novel retinamides (VNLG-152R, VNHM-1-81, VNHM-1-66, VNHM-1-73)	MNK degraders	Prostate and breast cancer	[84,85,86,87,88]
Galeterone and galeterone analogs	MNK degraders	Prostate cancer, PDAC	[89,90,91]
Pyridine derivatives (4t, MNK7g, MNKI-1, MNKI-8e, MNKI-8i, MNKI-19, MNKI-85, MNKI-4, MNKI-57, 12j)	MNK inhibitors	Cervical, breast, colorectal, ovarian, pancreatic and prostate cancer, medulloblastoma, leukemia, myeloma, nasopharyngeal carcinoma	[64,75,92,93,94,95,96,97,98,99,100,101]
SEL201 = SLV-2436	MNK inhibitor	Breast cancer, leukemia, melanoma	[102,103,104,105]
NUCC-54139	MNK inhibitor	Leukemia	[106]
Niclosamide (anthelmintic drug)	Targets ERK/MNK1/eIF4E	Leukemia	[76]
eFT508	MNK inhibitor	Lymphoma	[107]
QL-X-138	BTK/MNK inhibitor	Leukemia, lymphoma	[108]
FL3 (synthetic flavagline)	Ligand of PHBs (Targets ERK/MNK/eIF4E)	Lymphoma	[109]
Cyclopropanecarboxamide derivatives (53,54)	MNK inhibitors	Leukemia	[110]
Cabozantinib	Multi-kinase inhibitor	MPNSTs	[111]
apMNK2F, apMNK3R (aptamer)	MNK inhibitor	Breast cancer	[112]

Note: NSCLC—Non-small cell lung cancer; PCNSL—Primary central nervous system lymphoma; PDAC—Pancreatic ductal adenocarcinoma; MPNSTs—Malignant peripheral nerve sheath tumors.

**Table 2:** MNK inhibitors in clinical trials.

Compound	Phase	Name and Identifier	Status	Type of Cancer	Combination
BAY1143269	I	Phase I Dose Escalation and Expansion of Oral BAY1143269 in Combination With Intravenous Docetaxel NCT02439346	Terminated	Metastatic solid tumors	Docetaxel
eFT508 (Tomivosertib)	II	An Open-label Study Examining the Effect of Tomivosertib (eFT508) in Patients With Advanced Castrate-resistant Prostate Cancer (CRCP) NCT03690141	Active, not recruiting	Castrate-resistant prostate cancer	
	II	A Study to Evaluate eFT508 Alone and in Combination With Avelumab in Subljects With MSS Colorectal Cancer NCT03258398	Completed	Refractory colorectal cancer	Avelumab
	II	A PD Study of Oral eFT508 in Subjects With Advanced TNBC and HCC NCT03318562	Terminated	Triple negative breast cancer and hepatocellular carcinoma	
	I-II	A Dose Escalation and Cohort-Expansion Study of Oral eFT508 in Subjects With Advanced Solid Tumors NCT02605083	Terminated	Solid tumors	
	I-II	A Phase 1–2 Dose-Escalation and Cohort-Expansion Study of Oral Tomivosertib (eFT508) in Subjects With Hematological Malignancies NCT02937675	Terminated	Lymphoma	
	II	Tomivosertib (eFT508) in Combination With PD-1/PDL-1 Inhibitor Therapy NCT03616834	Active, not recruiting	Solid tumors	PD-1/PD-L1
	I	Safety, Pharmacodynamics, Pharmacokinetics, and Efficacy of Tomivosertib Combined With Paclitaxel in Advanced Breast Cancer NCT04261218	Not yet recruiting	Advanced breast cancer	Paclitaxel
Merestinib	I	Combination Merestinib and LY2874455 for Patients With Relapsed or Refractory Acute Myeloid Leukemia NCT03125239	Recruiting	Relapsed and Refractory Adult Acute Myeloid Leukemia	LY2874455
	I	A Study of Merestinib (LY2801653) in Japanese Participants With Advanced or Metastatic Cancer NCT03027284	Active, not recruiting	Advanced cancer, metastatic cancer, biliary tract carcinoma, cholangiocarcinoma, gall bladder carcinoma, solid tumor, non-Hodgkin’s lymphoma	Cisplatin and Gemcitabine
	I	Merestinib on Bone Metastases in Subjects With Breast Cancer NCT03292536	Recruiting	Bone metastases, breast cancer	
	I	A Study of Merestinib (LY2801653) in Healthy Participants NCT02779738	Completed		
	II	Merestinib in Non-Small Cell Lung Cancer and Solid Tumors NCT02920996	Active, not recruiting	Non-small cell lung cancer, solid tumors	
	II	A Study of Ramucirumab (LY3009806) or Merestinib (LY2801653) in Advanced or Metastatic Biliary Tract Cancer NCT02711553	Active, not recruiting	Biliary tract cancer, metastatic cancer, advanced cancer	Cisplatine and Gemcitabine
	I	A Study in Advanced Cancers Using Ramucirumab (LY3009806) and Other Targeted Agents NCT02745769	Completed	Advanced cancer, colorectal cancer, mantle cell lymphoma	Ramucirumab
	I	A Study of Anti-PD-L1 Checkpoint Antibody (LY3300054) Alone and in Combination in Participants With Advanced Refractory Solid Tumors NCT02791334	Recruiting	Solid tumor, microsatellite instability—high (MSI-H) solid tumors, cutaneous melanoma, pancreatic cancer, breast cancer (HR+HER2-)	LY3300054
Galeterone	II	1911GCCC: Galeterone or Galeterone With Gemcitabine for Patients With Metastatic Pancreatic Adenocarcinoma NCT04098081	Recruiting	Advanced Pancreatic Cancer	Gemcitabine
	I	Single-Dose Study to Assess the Absorption, Metabolism, Excretion, and Mass Balance of Radiolabeled Galeterone NCT02729376	Completed	Healthy	
	III	A Study of Galeterone Compared to Enzalutamide In Men Expressing Androgen Receptor Splice Variant-7 mRNA (AR-V7) Metastatic CRPC (ARMOR3-SV) NCT02438007	Terminated	Prostate cancer	
	II	A 2 Part Phase 2 Trial of Galeterone in the Treatment of Castration Resistant Prostate Cancer (ARMOR2) NCT01709734	Completed	Prostate cancer	

## Abbreviations

5’-UTR	untranslated region
AKT	also known as protein kinase B
ALL	Acute lymphocytic leukemia
AML	Acute myeloid leukemia
AR	androgen receptor
ARE	3’UTR regions of mRNA rich in residues A and U
ATO	arsenic trioxide
CML	chronic myeloid leukemia
CPLA2	cytoplasmic phospholipase A2
CRC	colorectal Cancer
CRPC	castration-resistant prostate cancer
DLBCL	Diffuse large B cell lymphoma
eIF	eukaryotic initiation factor
EMT	epithelial-mesenchymal transition
ERK	extracellular signal-regulated kinase
FGF	fibroblast growth factor
GBM	glioblastoma multiforme
HCC	hepatocellular carcinoma
HDM2	human double minute 2 homolog
hnRNP A1	heterogeneous nuclear ribonucleoprotein A1
IBC	inflammatory breast cancer
IL	interleukin
IRES	internal ribosomal entry sites
MAPK	mitogen-activated protein kinase
MCL-1	myeloid cell leukemia 1
MMP	metalloproteases
MNK	MAPK interacting kinase
MPNST	malignant peripheral nerve sheath tumor
mTOR	mammalian target kinase protein of rapamycin
NES	nuclear export signal
NLS	nuclear localization signal
NPC	nasopharyngeal carcinoma
NSCLC	non-small cell lung cancer
ODC	ornithine decarboxylase (ODC)
PCa	Prostate cancer
PCNSL	Primary central nervous system lymphoma
PDAC	Pancreatic ductal adenocarcinoma
PDX	patient-derived xenograft
PI3K	phosphatidylinositol-3 kinase
PSF	polypyrimidine tract-binding protein-associated splicing factor
PTEN	phosphatase and tensin homolog
RCC	renal cell carcinoma
SCLC	small-cell lung cancer
Spry2	sprouty 2
TCGA	the cancer Genome Atlas
TGFβ	Transforming growth factor β
TMZ	Temozolamide
TNBC	triple-negative breast cancer tumors
TNF	tumor necrosis factor
TOP	terminal oligopyrimidine
VEGF	vascular endothelial growth factor
WHO	World Health Organization
